# Adoption of new drugs by physicians: a survival analysis

**DOI:** 10.1186/1472-6963-12-56

**Published:** 2012-03-08

**Authors:** Francisco Javier Garjón, Ana Azparren, Iván Vergara, Borja Azaola, Jose Ramón Loayssa

**Affiliations:** 1 Servicio Navarro de Salud. Servicio de Prestaciones Farmacéuticas, Plaza de la Paz s/n, E-31002 Pamplona, Spain; 2 Servicio Navarro de Salud. Centro de Salud de Lodosa, Lodosa, Spain; 3 Servicio Navarro de Salud. Sección de Evaluación y Calidad en Atención Primaria, Pamplona, Spain; 4 Servicio Navarro de Salud. Centro de Salud de Huarte, Huarte, Spain

**Keywords:** Diffusion of innovation, Drug prescriptions, Drug utilization, Physician's practice patterns, Survival analysis

## Abstract

**Background:**

New drugs often substitute others cheaper and with a risk-benefit balance better established. Our aim was to analyse the diffusion of new drugs during the first months of use, examining the differences between family physicians and specialists.

**Methods:**

Prescription data were obtained of cefditoren, duloxetine, etoricoxib, ezetimibe, levocetirizine, olmesartan, pregabalin and tiotropium 36 months after their launching. We obtained the monthly number of prescriptions per doctor and the number prescribers of each drug by specialty.

After discarding those with less than 10 prescriptions during this period, physicians were defined as adopters if the number of prescriptions was over the 25th percentile for each drug and level (primary or secondary care). The diffusion of each drug was studied by determining the number of adopter family physicians throughout the study period. Among the group of adopters, we compared the month of the first prescription by family physicians to that of other specialists using the Kaplan-Meier method.

**Results:**

The adoption of the drugs in primary care follows an exponential diffusion curve that reaches a plateau at month 6 to 23. Tiotropium was the most rapidly and widely adopted drug. Cefditoren spread at a slower rate and was the least adopted. The diffusion of etoricoxib was initially slowed down due to administrative requirements for its prescription. The median time of adoption in the case of family physicians was 4-6 months. For each of the drugs, physicians of a specialty other than family physicians adopted it first.

**Conclusions:**

The number of adopters of a new drug increases quickly in the first months and reaches a plateau. The number of adopter family physicians varies considerably for different drugs. The adoption of new drugs is faster in specialists. The time of adoption should be considered to promote rational prescribing by providing timely information about new drugs and independent medical education.

## Background

Drugs are an essential part of medical practice and represent a high economic burden. The pattern of prescription varies among physicians and many influences on the doctor's prescription other than the clinical indications have been described [1-3]. In this context, the introduction and diffusion of new drugs on the market is an issue of special transcendence. The diffusion of a new drug can be defined as its dissemination through different channels over time among physicians [4].

The substitution of established therapies for new drugs is often inappropriate because of lack of improvements in effectiveness, when unknown side effects cannot be ruled out at the time of marketing [5] and the prices are disproportionate to the alleged benefits [6,7]. However, a delayed uptake of effective innovations supported by clinical investigation has also been observed, resulting in non-optimal treatments for some patients [8].

There is a substantial difference in prescribing a new drug for the first time ever and to prescribe it routinely [9]. A physician is an adopter of a new drug if he incorporates it to his personal formulary and hence prescribes it regularly.

Our study aims to analyze the diffusion of eight new drugs during the first months of use and examine the differences in the adoption of these new drugs between family physicians and specialists. We focus in adopters because we are interested in doctors who have changed their prescribing behaviour. The dynamics of this process is important to design educational or administrative activities.

Even though this study cannot identify all the factors that influence the diffusion of these drugs, it may be useful for generating hypothesis about the characteristics of the drugs that influence its diffusion and the relationship with the medical specialty. All this information could be used for establishing the timing of dissemination of scientific information on new drugs and for identifying sectors of doctors where a special attention would be required.

## Methods

We performed a retrospective study with the prescription database of the Navarre Health Service (*Servicio Navarro de Salud - Osasunbidea*, SNS-O) using data from 2003 to 2007. This database includes monthly information of all prescriptions financed by the SNS-O. By Spanish law, the aggregate information resulting from processing prescriptions of the National Health System is of public domain and its evaluation is competence of health services [10]. In Navarre the task belongs to the Pharmaceutical Benefits Service (*Servicio de Prestaciones Farmacéuticas*), which is part of the SNS-O. The Pharmaceutical Benefits Service provides feed back information to doctors about the use of new medicines, so the name of the doctor is included in the database.

Ethics statement: The study was approved by the scientific council of the Health Department of the Government of Navarre. We used a database that did not include patient information. Therefore, no ethical approval was necessary.

The SNS-O provides tax-financed health care in Navarre (Spain) and it covers pharmaceutical benefits to approximately 600,000 persons (95% of the population). Doctors working for the SNS-O are employees of it. All of them work in group practices or in hospitals. Specialists normally write the first prescription of the drug they indicate.

From the drugs marketed between 2003 and 2007, eight drugs with different indications were chosen (Table 1). All of them are suitable for use both at primary and at secondary care levels and were indicated in common disorders in clinical practice. During the study period those drugs were the latest licensed of their therapeutic group for their indications. The Drug Assessment Working Group of the SNS-O evaluates the degree of therapeutic innovation of the new drugs and provides drug assessment reports to the doctors belonging to the SNS-O. The degree of therapeutic innovation of each new drug is determined in accordance with criteria of level of evidence, efficiency, safety, convenience and cost, all in comparison with alternative therapies.

**Table 1:** Characteristics of the studied drugs

Drug	Launching	Degree of therapeutic Innovation*	Approved indications	Cost/DDD	Alternatives (cost/DDD)
**Cefditoren**	Sep-04	No therapeutic innovation	Pneumonia, exacerbation of chronic bronchitis, pharyngitis, tonsillitis, skin infections	€4.47 -6.26	Amoxicillin-clavulanate (€0.68) Cefuroxime axetil (€2.27)

**Duloxetine**	Dec-05	No therapeutic innovation	Neuropathic pain, depresión, generalised anxiety disorder (from jul-08)	€1.99	Amitriptyline (€0.11) Fluoxetine (€0.24) Paroxetine (€0.79)

**Etoricoxib**	Jul-04	No therapeutic innovation	Osteoarthritis, rheumatoid artritis, acute gouty artritis, ankylosing spondylitis (from sep-08)	€1.74	Ibuprofen (€0.24) Diclofenac (€0.17) Naproxen (€0.38)

**Ezetimibe**	Mar-04	Insufficient evidence	Primary hypercholesterolaemia, homozygous familial hypercholesterolaemia, homozygous sitosterolaemia	€1.91	

**Levocetirizine**	Apr-03	No therapeutic innovation	Allergic rhinitis, chronic idiopathic urticaria	€0.56	Cetirizine (€0.29)

**Olmesartan**	May-04	No therapeutic innovation	Hypertension	€0.92	Losartan (€0.92) Enalapril (€0.13)

**Pregabalin**	Jan-05	No therapeutic innovation	Neuropathic pain, epilepsy, generalised anxiety disorder (from mar-06)	€2.57	Gabapentin (€0.19) Amitriptyline (€0.11)

**Tiotropium**	Jan-03	Modest therapeutic innovation	Chronic obstructive pulmonary disease	€1.91	Ipratropium (€0.28)

*No therapeutic innovation: The new drug has no added value over other drugs which are already available in the market for the same indication. Modest therapeutic innovation: The new drug provides more posology comfort. Insufficient evidence: Available evidence is insufficient or inconclusive, or lacks good quality clinical trials including an adequate comparative drug.

*DDD *defined daily dose.

Information provided in the reports of the Navarre Drug Assessment Working Group [Available in: http://www.navarra.es/home_es/Temas/Portal+de+la+Salud/Profesionales/Documentacion+y+publicaciones/Publicaciones+tematicas/Medicamento/BIT/].

In the case of etoricoxib, a prior authorization was required for its prescription at the onset of marketing. This requirement was withdrawn 24 month after its launching.

For each drug, the month in which the first prescription was issued was considered month 1 and a follow up of the drug prescription was carried out for a period of up to 36 months.

Doctors who had signed some prescription in 2003 and also in 2007 were eligible.

We select those who prescribed any of the eight studied drugs. From this group we analyzed the number of prescriptions per physician at months 2, 3, 6, 12, 24 and 36 in family physicians.

To define the group of adopters of a drug, we first excluded those physicians who prescribed it sporadically (less than 10 prescriptions during the 36 month study period). Then for each drug and level (primary care or secondary care) we selected physicians whose number of prescriptions was over the 25th percentile of prescriptions per drug and physician within each level. Using this criterion we deal with the differences in the number of patients, contacts and case-mix.

The diffusion of each drug was studied among the group of adopter physicians. The adoption time of a drug was defined as the month in which the physician makes the first prescription. We plotted the curve of cumulative number of adopter primary care physicians over time after the launching of the drug. A Kaplan-Meier survival analysis was performed with the adoption time. The log rank test was used to test differences between family physicians and the different specialists. Statistical analysis was performed using the program PASW Statistics^® ^for Windows (version 17.0, SPSS Inc.).

## Results

**Figure 1:**
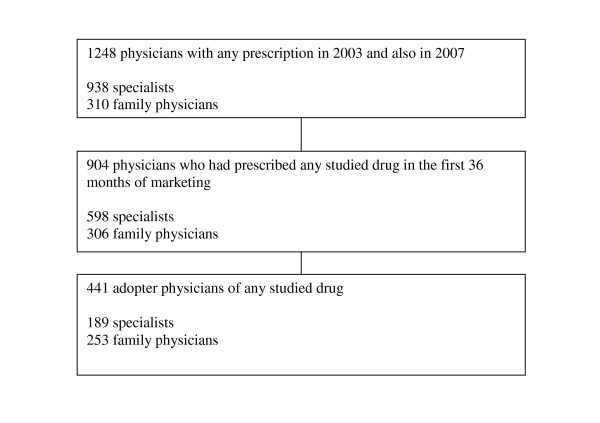
**Flow diagram**.

Of 1248 physicians with a prescription, 904 prescribed some of the selected drugs and 441 were considered as adopters (Figure 1).

### Prescribing family physicians and mean of prescriptions

Table 2 shows the number of family physicians who have prescribed each studied drug and the mean of prescriptions per physician at different times.

**Table 2:** Family physicians who prescribe each drug at different times and mean prescriptions per physician

	Month since launching											
	
	2		3		6		12		24		36	
	
	n	*Mean (SD)*	n	*Mean (SD)*	n	*Mean (SD)*	n	*Mean (SD)*	n	*Mean (SD)*	n	*Mean (SD)*
**Cefditoren**	7	3.6 *(4.2)*	26	2.3 *(1.9)*	30	3.4 *(2.8)*	32	3.3 *(5.7)*	44	2.0 *(2.2)*	51	2.6 *(2.8)*

**Duloxetine**	38	1.5 *(0.9)*	67	2.0 *(1.5)*	125	2.4 *(2.0)*	172	3.5 *(2.7)*	176	5.1 *(3.8)*	*	*

**Etoricoxib**	14	1.1 *(0.4)*	32	1.6 *(0.9)*	41	1.4 *(0.6)*	44	1.5 *(0.8)*	48	1.9 *(1.3)*	124	3.1 *(4.0)*

**Ezetimibe**	31	2.0 *(1.4)*	59	2.0 *(1.5)*	94	2.3 *(1.9)*	148	2.7 *(2.5)*	200	4.0 *(3.6)*	191	4.6 *(3.8)*

**Levocetirizine**	8	1.6 *(0.9)*	9	1.6 *(0.7)*	21	1.2 *(0.4)*	75	2.2 *(1.6)*	132	2.4 *(1.7)*	158	3.0 *(2.8)*

**Olmesartan**	36	1.8 *(1.0)*	49	2.2 *(1.3)*	89	2.3 *(1.7)*	131	3.4 *(3.7)*	165	4.1 *(4.8)*	182	4.7 *(5.5)*

**Pregabalin**	14	1.3 *(0.5)*	31	1.7 *(1.4)*	80	1.8 *(1.0)*	137	2.3 *(1.8)*	181	3.7 *(2.7)*	198	5.1 *(3.6)*

**Tiotropium**	80	2.3 *(1.6)*	127	2.9 *(2.4)*	215	3.5 *(2.6)*	244	5.9 *(4.0)*	264	7.4 *(4.9)*	264	8.5 *(5.4)*

*n *number of physicians. *SD *standard deviation.

*Data of 36^th ^month of duloxetine are no comparable due to changes in medical staff.

### Rate of adoption of the drugs among physicians in each specialty

**Figure 2:**
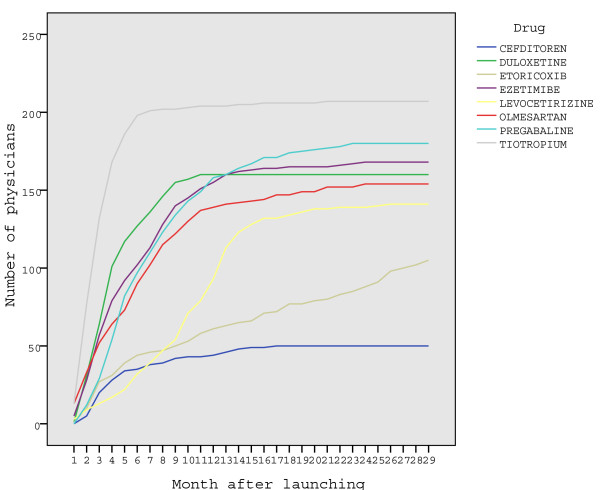
**Cumulative number of adopter family physicians who prescribe each drug over time**.

The diffusion of drugs among adopter family physicians is presented in Figure 2.

The adoption of the new drugs under study in primary care shows a similar pattern, represented by an exponential diffusion curve [11]. In the first months after the launching of the drug, the number of adopters increased rapidly to finally reach a plateau. Curves show different slopes (speed of adoption) and different heights (number of doctors who eventually adopted the drugs). The curves reach plateaus in different months, between 6 and 23, reflecting different rates of diffusion of the drugs.

Tiotropium was the most rapidly disseminated and most widely adopted drug. Cefditoren spread at the slowest rate and was the least adopted. Etoricoxib was an exception because it did not reach a plateau during the study period.

The differences between specialties in adoption of the drugs under study are shown in Table 3. Although each drug is prescribed by several specialties, the proportion of adopters is very different. For each drug there is one specialty that adopted it before family physicians and with a percentage of adopters of at least 50%: otolaryngology for cefditoren, psychiatry for duloxetine, rheumatology for etoricoxib, endocrinology for ezetimibe, allergology for levocetirizine, rheumatology for pregabalin and pneumology for tiotropium. Olmesartan was an exception since the percentage of family physician adopters was far larger than specialists although cardiology adopted it faster.

**Table 3:** Adopters per drug and specialty and time of adoption

				Time of adoption		
				
Drug	Specialty	Number of prescribers	Number of adopters (%)	Median in months (intercuartile range)	Comparison with Family Physicians. Log-rank test	
					
					**χ**^2^	p-value
**Cefditoren**	Otolaryngology	19	11 (58%)	2 (2-5)	6.76	0.01
	
	*Family physicians*	*232*	*50 *(22%)	*4 (3-7)*		
	
	Internal medicine	22	4 (18%)	5 (3-10)	0.44	NS
	
	Emergencies	65	7 (11%)	9 (5-21)	6.39	0.01
	
	Pneumology	15	7 (47%)	9 (8-12)	1.14	NS

**Duloxetine**	Psychiatry	36	22 (61%)	2 (2-3)	29.55	< 0.01
	
	*Family physicians*	*252*	*160 *(63%)	*4 (3-6)*		

**Etoricoxib**	Rheumatology	6	3 (50%)	2 (1-8)	8.08	< 0.01
	
	Traumatology	55	21 (38%)	3 (2-15)	1.97	NS
	
	*Family physicians*	*230*	*105 *(46%)	*10 (3-20)*		
	
	Rehabilitation	14	2 (14%)	11 (11-26)	0.51	NS

**Ezetimibe**	Cardiology	16	6 (38%)	2 (1-3)	8.13	< 0.01
	
	Endocrinology	11	8 (73%)	3 (2-3)	10.95	< 0.01
	
	*Family physicians*	*258*	*168 *(65%)	*5 (3-8)*		

**Levocetirizine**	Otolaryngology	19	8 (42%)	1 (1-4)	24.14	< 0.01
	
	Allergology	10	10 (100%)	4 (2-7)	4.96	0.03
	
	Emergencies	44	4 (9%)	4 (4-12)	0.73	NS
	
	Dermatology	15	12 (80%)	9 (2-12)	1.54	NS
	
	*Family physicians*	*264*	*141 *(53%)	*10 (7-13)*		

**Olmesartan**	Cardiology	15	3 (20%)	1 (1-2)	17.23	< 0.01
	
	Internal medicine	21	3 (14%)	2 (2-13)	0.04	NS
	
	*Family physicians*	*256*	*154 (60%)*	*6 (3-9)*		

**Pregabalin**	Anesthesiology	8	3 (38%)	2 (2-5)	8.45	< 0.01
	
	Neurology	17	8 (47%)	3 (2-4)	12.1	< 0.01
	
	Rheumatology	6	6 (100%)	3 (2-4)	9.83	< 0.01
	
	Rehabilitation	23	17 (74%)	5 (2-7)	2.94	NS
	
	*Family physicians*	*275*	*180 *(65%)	*6 (4-10)*		
	
	Traumatology	56	17 (30%)	6 (4-9)	0.87	NS

**Tiotropium**	Pneumology	15	14 (93%)	1 (1-2)	49.18	< 0.01
	
	Internal medicine	30	14 (47%)	2 (2-5)	0.13	NS
	
	*Family physicians*	*297*	*207 *(70%)	*3 (2-4)*		

NS: *p *> 0.05

## Discussion

The adoption of new drugs in primary care follows an exponential curve. In consumer research, this shape indicates consumers perceive little risk (physical or economic) associated with the innovation [11]. We believe this perception is not desirable in the field of new drugs.

The adoption rate of a drug can have been influenced by several factors apart from how effectively it is marketed.

### Degree of therapeutic innovation

It is determined in accordance with criteria of evidence, efficiency, safety, convenience and cost, all in comparison with alternative therapies. Tiotropium was the most widely and quickest adopted drug by family physicians. It was the only studied drug that was rated as a therapeutic innovation but we cannot affirm that it is the main reason for its rapid diffusion. In the literature, however, this quality is not related consistently with adoption [12,13]. Personal perceptions about effectiveness, safety profile and advantages over alternatives are consistent factors that influence the decision to adopt a drug [14,15]. Cost does not seem to be a determining factor [3,16].

### Me-too drugs

They are drugs chemically related with a previous approved drug, with the same mechanism of action and indications. Since there is a perception of well known drugs, its adoption can be facilitated. On the other hand, there are few reasons for adopting them due to their scarce therapeutic advantages. Olmesartan belongs to a commonly used class of drugs, angiotensin II receptor blockers, employed in the management of hypertension. Although it was not massively adopted, the percentage of adopters was greater for family physicians than for specialists. It can be explained by their indication which is managed almost entirely in primary care.

Levocetirizine, a isomer of cetirizine, is an example of strategy to prolong the life of drug when the patent expires in the face of competition from generic drugs [17].

### New mechanism of action

Doctors are likely to prescribe drugs with a new mechanism of action [3]. Ezetimibe has the same indications than effective therapies like statins; but its mechanism of action is different. The decreasing lipid targets in guidelines may have promoted its adoption as add-on therapy.

### Range of indications

Pregabalin was adopted initially at a not very fast rate, but at the end of the study period was adopted by a large number of physicians. This may be because the range of its indications was increased. Besides, pregabalin is indicated for diseases with a poor response to therapies like neuropathic pain or generalised anxiety disorder.

Duloxetine has the indication of depression, a very common condition that explains the wide adoption for psychiatrists and for family physicians. Surprisingly, although it is also indicated in neuropathic pain, in contrast with pregabalin, it was not adopted by the specialties that deal with this disease (anaesthesiology, neurology and rheumatology).

### Policies

Cefditoren had the lower number of adopters in primary care. The policy of rational use of antibiotics may have limited its utilization.

Etoricoxib initially required an endorsement before dispensation, which was subsequently withdrawn allowing for wider prescription henceforth. The effect of an authorization requirement for COX-2 inhibitor drugs has been determined in the U.S.A [18].

### Chronic versus acute use

Due to the use of ceftditoren in acute diseases, there is not induced prescription (treatment initiated in secondary care and followed in primary care). This can have contributed to its low adoption.

The seasonal use of levocetirizine can be responsible for its long adoption time.

The results from this study suggest that secondary care plays a key role in the adoption of new drugs by family physicians. Drugs are adopted earlier by specialists. This fact could be justified when the drugs are indicated in a disease managed mainly by specialists. The early adopter role of the specialists may be due to various factors: differences in sources of information [3,16], differences in the attitude towards evidence-based medicine [19] and differences in the tolerance to uncertainty [20].

Specialists' prescription influence family physicians by induced or imitated prescriptions [3,21]. Our study can not differentiate between prescriptions originating from primary care and induced.

Why some specialties have a low percentage of adopters, even lower than family physicians? Specialists always choose the drugs they use. However, the family physician often follows the prescriptions of the specialists. Another reason can be the existence of sub-specialties (e.g. a neurologist who treats mainly Parkinson disease can use few antiepileptic drugs).

This study focuses on adopters because our interest is the process of inclusion of a drug in the therapeutic arsenal of physicians and not the occasional prescription. The definition of an adopter of a new drug was *ad hoc*. We do not categorise physicians as early or late adopters, as is common in the literature about diffusion of new drugs [4,12,22]. The notion that early adoption is a personal characteristic has been challenged [9].

We use time-to-event analysis to compare adoption times between primary and secondary care. We find this model useful to describe the process of adoption because time is a key variable for designing intervention strategies. Another study used a Cox model to compare single-handed to partnership practices [9].

This study has several limitations. The analysis is limited to eight drugs and the results may not be generalized. We could not differentiate between prescriptions originating from primary care and those induced.

The median time of adoption for family physicians is between 4 and 6 months for the majority of the drugs. This period is shorter for specialists. New drugs are adopted before safety and cost-effectiveness are well established. At the time a new drug reaches the market, information about its efficacy and safety came only from clinical trials. Generally, they are too short, with too few patients and too narrow. Only selected patients are included. Those older, non compliant, with polypharmacy or with multiple pathologies are often excluded. This makes impossible the accurate identification of adverse effects. The selection of patients in clinical trials and the reliance in subrogate end points prevent from establishing the effectiveness in real life situations and hence the cost- effectiveness.

"Do Not Rush to Use Newly Marketed Drugs" has been proposed as a principle of judicious prescribing. Older drugs are generally safer owing to their longer track record. Even, to wait 7 years before using a new drug has been advocated, based on data showing that it often takes 5 to 10 years to identify significant adverse effects [23]. This is in great contrast with the adoption rate in our study.

New drugs often replace others better known and cheaper. The short period of adoption should be taken into account if we consider the implementation of measures aimed at controlling the diffusion of a new drug (e.g. independent drug information or medical education activities). Albeit there is a large room for improvement in drug regulatory agencies [23], health systems have the responsibility to design strategies to promote the cautious adoption of innovative drugs; inhibit the adoption of the non-innovative ones; and avoid the premature abandonment of established therapies. If there is a risk of a delayed introduction of truly innovative products has to be balanced against the risk of treating patients with a drug before its safety was well established.

Ideally, information about the relative efficacy and safety of a new drug should be known before launching or at least before being adopted. This information has to be evidence-based and independent of the pharmaceutical industry. Drug bulletins publish reviews of new drugs trying to help their readers recognise the products that really are an advance and which deserve to be included in the list of drugs they use. Outreach visits, continuing education meetings and workshops are also used. All those instruments show limited effectiveness. Multifaceted interventions are more effective than simply delivering information [24].. It is necessary to consider that interventions are often carried out against aggressive marketing campaigns of the pharmaceutical industry that can promote inappropriate prescription [25]. Therefore, it should be recognised that modifying prescribing behaviour is difficult. The design of interventions should have a wider scope taking into account the attitudes and beliefs of doctors, and the existence of professional networks both formal and informal [3,16,19]. The imposition of administrative requirements could be effective.

It is necessary to explore the influence of factors such as the relationship with the pharmaceutical industry, continuing education and independent drug information. Moreover, the identification of other factors could help explain the dynamics of new drugs adoption in order to design better strategies for promoting the selection of cost-effective therapies.

## Conclusions

The number of adopters of a new drug increases quickly in the first months and reaches a plateau. The number of adopter family physicians varies considerably for different drugs. The adoption of new drugs is faster in specialists. The time of adoption should be considered to promote rational prescribing by providing timely information about new drugs and independent medical education.

## Competing interests

IV has received payment for educational presentations from Novartis and Esteve. BA has received payment for educational presentations from GSK and reimbursement of travel expenses from MSD and Pfizer. JRL has received payment for educational presentations from Novartis and reimbursement of travel expenses from Esteve, Novartis and Pfizer. Both FJG and AA declare that they have no conflict of interest.

## Authors' contributions

All authors participated in the design of the study and the discussion of findings. FJG and AA executed the data management. JRL drafted the manuscript. FJG, AA, IV and BA revised the manuscript. All authors read and approved the final manuscript.

## Pre-publication history

The pre-publication history for this paper can be accessed here:

http://www.biomedcentral.com/1472-6963/12/56/prepub
